# Risk factors and a predictive model for delayed extubation in children undergoing primary repair of tetralogy of Fallot: a retrospective cohort study with rigorous methodological validation

**DOI:** 10.3389/fped.2026.1902972

**Published:** 2026-08-03

**Authors:** Mingwei Wang, Shumin Wu, Kexue Wang, Kun Li, Zhenyu Wu, Liwei Yan, Qihui Shen, Faming He

**Affiliations:** 1 Department of Cardiovascular and Thoracic Surgery, Henan Chest Hospital, Zhengzhou University, Zhengzhou, China; 2 Cardiac Surgery Intensive Care Unit, Henan Chest Hospital, Zhengzhou University, Zhengzhou, China

**Keywords:** bootstrap validation, decision curve analysis, delayed extubation, LASSO regression, nomogram, predictive model, risk factors, tetralogy of Fallot

## Abstract

**Objective:**

To identify independent risk factors for delayed extubation (mechanical ventilation 48 h) following primary Tetralogy of Fallot (TOF) repair and to develop a rigorously validated predictive model for clinical use.

**Methods:**

A retrospective cohort study was conducted involving 189 children treated at Henan Chest Hospital between 2019 and 2023. Patients were stratified into delayed (*n* = 101) and non-delayed (*n* = 88) extubation groups. The analytical pipeline incorporated Shapiro–Wilk testing, univariate screening, and LASSO regression with 10-fold cross-validation for variable selection. Multicollinearity was assessed via Variance Inflation Factor (VIF), followed by multivariate logistic regression. Model performance was comprehensively evaluated using ROC curves, calibration plots with the Hosmer–Lemeshow test, and Decision Curve Analysis (DCA). Internal validation was secured through 1000-iteration bootstrap resampling and 10-fold cross-validation.

**Results:**

Six independent predictors were ultimately retained: inotropic score (OR = 1.999), oxygenation index (OR = 0.990), RVOT diameter (OR = 0.711), CPB time (OR = 1.027), McGoon ratio (OR = 0.114), and postoperative PVOV (OR = 1.947). The model demonstrated excellent discrimination with an AUC of 0.916 (bootstrap-corrected 0.909). Calibration was robust (Hosmer–Lemeshow *P* = 0.366, Brier score = 0.113), and DCA confirmed significant clinical net benefit across threshold probabilities. Sensitivity analysis highlighted the inotropic score as the most influential variable.

**Conclusions:**

This internally validated model integrates preoperative anatomical (McGoon ratio, RVOT diameter), intraoperative (CPB time), and postoperative physiological (inotropic score, oxygenation, PVOV) factors. It may serve as a potentially useful tool for early risk stratification and could support a dual-phase assessment strategy to guide targeted interventions in pediatric TOF repair. External validation in independent cohorts and prospective clinical implementation studies are warranted before routine clinical adoption.

## 1 Introduction

Tetralogy of Fallot (TOF) is the most prevalent cyanotic congenital heart disease (CHD) in pediatrics, comprising approximately 5%–10% of all congenital cardiac malformations (1, 2). The condition is anatomically characterized by the classic tetrad of malformations: pulmonary stenosis predominantly involving the infundibulum of the right ventricular outflow tract, a malalignment type ventricular septal defect, aortic override, and secondary right ventricular hypertrophy (3). This unique pathophysiology results in chronic hypoxemia, polycythemia, and compromised pulmonary blood flow, placing infants at significant risk for hypoxic spells and long-term neurodevelopmental sequelae in the absence of treatment (4, 5). While advances in surgical correction have markedly improved survival, the inherent invasiveness and length of the procedure contribute to considerable perioperative morbidity and mortality. These challenges persist despite continuous refinements in surgical technique (6, 7).

Although surgical correction achieves high success rates, the perioperative period remains fraught with challenges, particularly regarding respiratory management. The procedure necessitates cardiopulmonary bypass (CPB), which triggers a systemic inflammatory response leading to endothelial injury and capillary leak syndrome (8). These physiological disturbances, combined with the inherent vulnerability of the pediatric respiratory system—characterized by compliant chest walls, narrow airways, and limited respiratory reserve—create a perfect storm for pulmonary morbidity (9). A primary clinical endpoint reflecting this vulnerability is the duration of postoperative mechanical ventilation. While early extubation is a desirable goal associated with reduced rates of ventilator-associated pneumonia, shorter intensive care unit (ICU) stays, and decreased healthcare costs, achieving it remains difficult in a subset of patients (10, 11). Consequently, delayed extubation (commonly defined as mechanical ventilation exceeding 48 h) affects a significant proportion of TOF patients and serves as a critical marker for complicated recovery and heightened resource utilization (12, 13).

Therefore, understanding the precise determinants of delayed extubation in this specific cohort is paramount for refining perioperative strategies. Although previous studies have identified factors such as prolonged CPB time and low cardiac output syndrome as contributors to morbidity, there remains a paucity of robust, integrated predictive models that combine preoperative anatomical risk factors with dynamic postoperative physiological data (14, 15). Identifying these risk factors before and immediately after surgery could enable clinicians to stratify patients, anticipate complications, and implement targeted interventions to facilitate earlier liberation from mechanical ventilation (16, 17). This study aims to bridge this gap by retrospectively analyzing clinical data from children undergoing primary TOF repair, elucidating the independent risk factors for delayed extubation, and constructing a comprehensive predictive model to aid in the early identification of high-risk patients, ultimately seeking to optimize postoperative outcomes and reduce the burden of prolonged ventilation.

## 2 Methods

### 2.1 Study design and population

This retrospective cohort study included 189 consecutive children (Aged: 1.1 months to 132 months) who underwent primary TOF repair at Henan Chest Hospital between May 2019 and December 2023. The study protocol complied with medical ethics standards and was approved by the Hospital Ethics Committee (Ethical Approval No.: (2025) Ke Lun Shen No. (02-04)). Inclusion criteria were: (1) confirmed diagnosis of TOF by echocardiography and/or cardiac CT; (2) primary complete surgical repair; and (3) complete clinical data available. Exclusion criteria were: (1) previous palliative or corrective cardiac surgery; (2) concurrent major non-cardiac anomalies; and (3) intraoperative death or postoperative death prior to extubation (including ICU death).

With 189 patients and 101 delayed extubation events in the final model containing 6 predictors, the events-per-variable (EPV) ratio was approximately 16.8:1, exceeding the commonly recommended minimum threshold of 10 EPV for stable multivariate logistic regression coefficient estimation (35). For the initial pool of 19 postoperative variables screened by LASSO regression, the EPV ratio was 5.3:1, which is acceptable given LASSO's superior performance in high-dimensional settings with modest sample sizes. This sample size provides adequate power for reliable variable selection and stable model estimation.

### 2.2 Definition of outcome

The primary outcome was delayed extubation, defined as mechanical ventilation duration ≥48 h after TOF repair. This threshold was selected based on established literature defining prolonged mechanical ventilation in pediatric cardiac surgery and aligns with clinical decision-making regarding ventilator weaning strategies. Patients were accordingly stratified into the delayed extubation group (*n* = 101) and the non-delayed extubation group (*n* = 88).

#### 2.2.1 Ventilator weaning and sedation protocol

During the study period (2019–2023), our center maintained a consistent, protocol-driven approach to postoperative mechanical ventilation and weaning. The standardized ventilator weaning protocol follows a stepwise algorithm: (1) initiation of pressure support ventilation (PSV) mode once hemodynamic stability is achieved (mean arterial pressure appropriate for age, no escalating inotropic support); (2) daily spontaneous breathing trials (SBT) using pressure support of 8–10 cmH_2_O with positive end-expiratory pressure (PEEP) of 5 cmH_2_O; (3) assessment of extubation readiness using standardized criteria including adequate oxygenation (PaO_2_/FiO_2_ > 150), adequate ventilation (PaCO_2_ < 50 mmHg with pH > 7.30), hemodynamic stability without significant arrhythmias, and adequate cough reflex and airway patency; (4) extubation performed by the attending cardiac intensivist in collaboration with the cardiac surgeon. Sedation management follows a nurse-driven protocol using daily sedation interruptions with fentanyl and midazolam infusions, transitioning to dexmedetomidine when hemodynamically tolerated. Analgesia is provided with intravenous fentanyl boluses and acetaminophen. No fundamental changes were made to this protocol during the study period, ensuring temporal consistency in perioperative management.

### 2.3 Data collection and Variable classification

A comprehensive set of 46 clinical variables was collected, encompassing preoperative, intraoperative, and postoperative parameters. To avoid circular reasoning and ensure temporal validity of the predictive model, variables were strictly classified into predictors and outcome-related variables. The 37 candidate predictor variables included: (1) Preoperative factors (14 variables): gender, age, weight, cardiothoracic ratio, SpO_2_, hemoglobin, VSD diameter, RVOT diameter, preoperative PVOV, associated anomalies, hypoxic spells, McGoon ratio, Nakata index, and LVEDVI; (2) Intraoperative factors (4 variables): transannular patch use, operative time, aortic cross-clamp time, and CPB time; (3) Postoperative factors (19 variables): postoperative RV/PV pressure gradient, postoperative left and right atrial pressure, residual shunt, oxygenation index, pH, hematocrit, lactate, postoperative hemoglobin, postoperative PVOV, inotropic score, ALT, AST, total/direct/indirect bilirubin, BUN, creatinine, and ejection fraction. All postoperative variables were measured within the first 6 h after ICU admission to capture the earliest available postoperative physiological state. Eight outcome-related variables (ascites, pleural effusion, low cardiac output syndrome, arrhythmia, peritoneal dialysis, hospital length of stay, ICU length of stay, reintubation) were excluded from predictor analysis as they represent consequences of delayed extubation rather than antecedent risk factors. Extubation decisions were made by the attending intensivist based on our center's standard weaning protocol, which requires hemodynamic stability, adequate gas exchange, minimal inotropic support, and satisfactory spontaneous breathing effort.

### 2.4 Statistical analysis

All statistical analyses were performed using Python 3.12 with scipy 1.14.1, statsmodels 0.14.5, scikit-learn 1.5.2, and matplotlib 3.9.2. A two-sided *P* < 0.05 was considered statistically significant.

#### 2.4.1 Descriptive statistics

Shapiro–Wilk test was applied to all continuous variables to assess normality. As all variables demonstrated non-normal distribution (*P* < 0.05), continuous variables were expressed as median (interquartile range, IQR) and compared using Mann–Whitney *U*-test. Categorical variables were expressed as frequency (percentage) and compared using chi-square test or Fisher exact test as appropriate. Effect sizes were calculated for all comparisons: rank-biserial correlation (*r*) for continuous variables and Cramer V for categorical variables, providing a standardized measure of association magnitude independent of sample size.

#### 2.4.2 Univariate analysis

Univariate logistic regression was performed for each of the 37 candidate predictor variables. Variables with *P* < 0.10 in univariate analysis were considered for inclusion in subsequent multivariate modeling, using a more liberal threshold to avoid premature exclusion of potentially important predictors. Odds ratios (OR) with 95% confidence intervals were calculated for each variable.

#### 2.4.3 LASSO regression for variable selection

Least Absolute Shrinkage and Selection Operator (LASSO) regression was applied to the variables passing the univariate screen (*P* < 0.10). LASSO applies L1 regularization that shrinks coefficients of less important variables to exactly zero, effectively performing variable selection while accounting for correlations among predictors. Our three-stage variable selection approach (univariate screening → LASSO → backward elimination) leverages the complementary strengths of each method: univariate screening reduces dimensionality to prevent LASSO from being overwhelmed by noise; LASSO handles multicollinearity and selects the most parsimonious predictor set; backward elimination ensures all retained variables have statistically significant independent contributions with directly interpretable odds ratios. This sequential approach is well-established in clinical prediction model development and has been recommended in methodological guidelines. The optimal regularization parameter (lambda) was determined through 10-fold cross-validation using the 1-standard error rule (lambda.1se), which selects the most parsimonious model within one standard error of the minimum cross-validation error, balancing predictive accuracy with model simplicity. Variables with non-zero LASSO coefficients were advanced to multivariate modeling.

#### 2.4.4 Multicollinearity assessment

Variance inflation factor (VIF) was calculated for all variables selected by LASSO regression. VIF quantifies the degree to which the variance of an estimated regression coefficient increases due to multicollinearity. A VIF threshold of 5 was used, with values below this indicating acceptable multicollinearity. This step ensures that the multivariate model coefficients are stable and interpretable.

#### 2.4.5 Multivariate logistic regression

Variables identified by LASSO regression and confirmed by VIF analysis were entered into a multivariate logistic regression model using maximum likelihood estimation. Backward elimination was then applied, sequentially removing the variable with the highest *P*-value above 0.05 until all remaining variables were statistically significant. The final model was evaluated using likelihood ratio test, McFadden pseudo-R-squared, Akaike Information Criterion (AIC), and Bayesian Information Criterion (BIC).

#### 2.4.6 Model performance evaluation

Model discrimination was assessed using receiver operating characteristic (ROC) curve analysis with area under the curve (AUC). For individual predictors, ROC analysis was performed with directionality correction to ensure proper interpretation of variables where lower values indicate higher risk. Optimal cut-off values were determined using Youden index. Model calibration was evaluated through: (1) calibration plot comparing predicted vs. observed probabilities across deciles; (2) Hosmer–Lemeshow goodness-of-fit test (*P* > 0.05 indicating adequate calibration); and (3) Brier score (lower values indicating better calibration, with 0.25 representing a non-informative model for binary outcomes).

#### 2.4.7 Decision curve analysis

Decision curve analysis (DCA) was performed to evaluate the clinical utility of the predictive model. DCA calculates the net benefit of using the model across a range of threshold probabilities, comparing it against two default strategies: treating all patients (assume all delayed) and treating none (assume none delayed). A model demonstrates clinical value when its net benefit exceeds that of both default strategies across clinically relevant threshold probabilities. Clinical impact curves were also generated to visualize the number of patients classified as high-risk and the proportion of true positives at each threshold probability.

#### 2.4.8 Nomogram construction

A nomogram was constructed based on the final multivariate logistic regression model, providing a visual tool for individualized risk prediction. Each predictor variable is assigned a point scale proportional to its regression coefficient, and the total points are mapped to a predicted probability of delayed extubation, enabling clinicians to rapidly estimate individual patient risk at the bedside.

#### 2.4.9 Internal validation

Internal validation was performed using two complementary approaches: (1) Bootstrap resampling with 1,000 iterations: for each iteration, a bootstrap sample was drawn with replacement, the model was refit, and AUC was calculated on both the bootstrap sample and the original sample. The optimism was estimated as the average difference between bootstrap and original AUCs, and the corrected AUC was computed by subtracting optimism from the apparent AUC. The 95% confidence interval for AUC was derived from the bootstrap distribution. (2) Stratified 10-fold cross-validation: the dataset was divided into 10 folds maintaining the same proportion of delayed extubation cases in each fold, and the model was trained on 9 folds and tested on the held-out fold, with AUC calculated for each iteration.

#### 2.4.10 Subgroup and sensitivity analyses

Subgroup analyses were performed stratifying by age (>12 months vs. ≤12 months), weight (≤10 kg vs. >10 kg), and transannular patch use (with vs. without). Model discrimination (AUC) was calculated within each subgroup to assess model robustness across patient subpopulations. Sensitivity analysis was conducted using two approaches: (1) Leave-one-variable-out analysis, where each predictor was sequentially removed from the final model and the change in AUC was recorded, identifying the most influential predictors; and (2) analysis using different *P*-value thresholds (0.05, 0.10, 0.15, 0.20) for univariate variable inclusion to assess whether the final model was sensitive to the initial variable screening criterion.

#### 2.4.11 Missing data handling

Missing data were minimal across all candidate variables (<3% for any variable) due to the standardized electronic data collection protocol at our center. For the few cases with missing values, we applied multiple imputation by chained equations (MICE) with 10 imputed datasets, using all other candidate predictors and the outcome variable as predictors in the imputation model. Variables were imputed separately for the delayed and non-delayed extubation groups to preserve outcome-dependent distributions. The LASSO regression and multivariate modeling were then performed on each imputed dataset, and the final model estimates were pooled using Rubin's rules.

## 3 Results

### 3.1 Baseline characteristics

**Figure 1:**
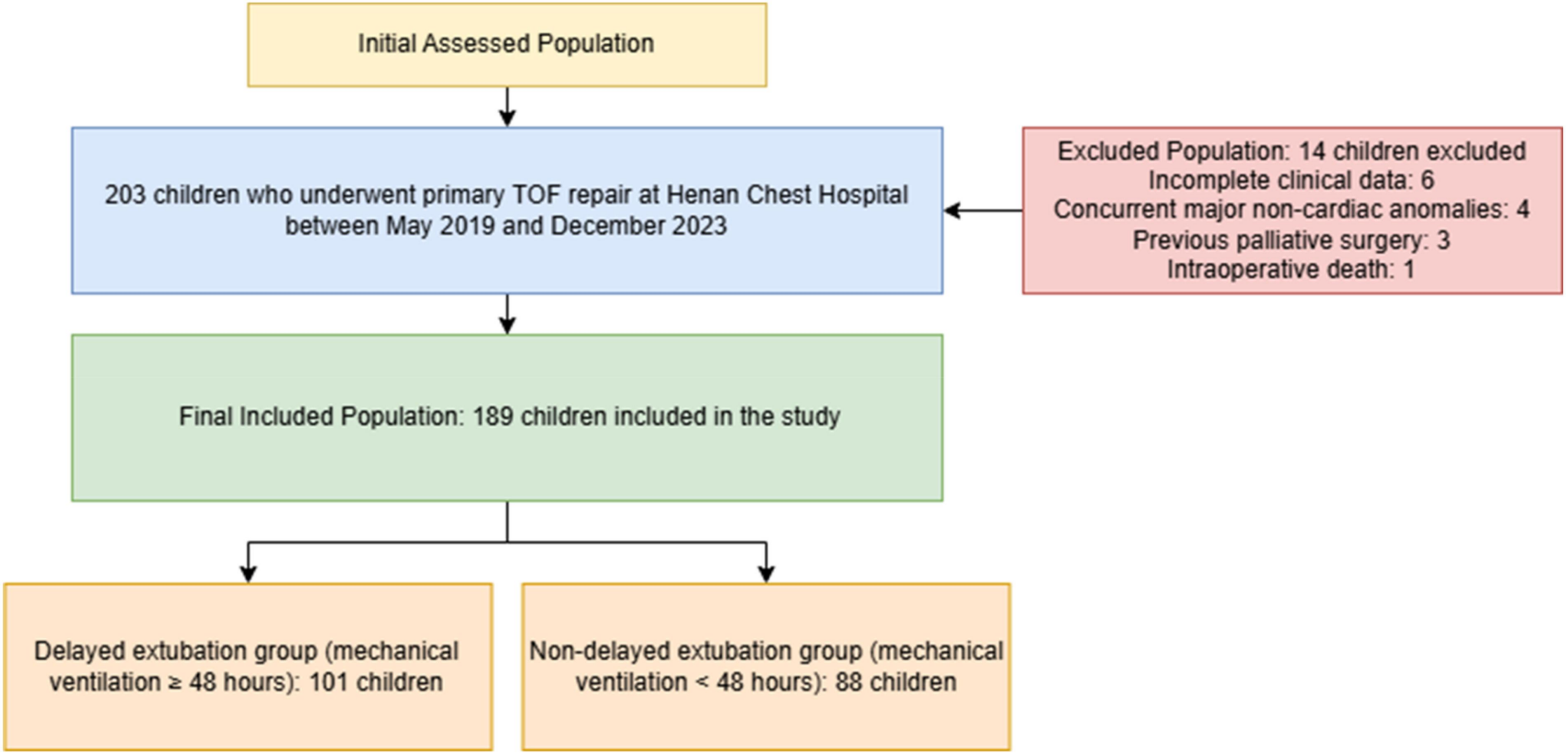
Participant flowchart of this study.

A total of 189 children who underwent primary TOF repair were included in this study, with 101 (53.4%) in the delayed extubation group and 88 (46.6%) in the non-delayed extubation group (Table 1). Of the 203 patients who underwent primary TOF repair during the study period, 14 were excluded: 6 with incomplete clinical data, 4 with concurrent major non-cardiac anomalies, 3 with previous palliative surgery, and 1 intraoperative death (Figure 1). Shapiro–Wilk testing confirmed that all continuous variables were non-normally distributed (all *P* < 0.05), supporting the use of non-parametric methods. Compared with the non-delayed group, the delayed extubation group had significantly lower SpO_2_ (median 89% vs. 94%, *P* = 0.007, *r* = 0.640), smaller RVOT diameter (4.10 mm vs. 5.00 mm, *P* < 0.001, *r* = 0.714), lower McGoon ratio (1.75 vs. 1.90, *P* = 0.002, *r* = 0.655), lower Nakata index (194 vs. 252, *P* < 0.001, *r* = 0.668), longer CPB time (57 min vs. 48 min, *P* < 0.001, *r* = 0.372), lower oxygenation index (246 vs. 310, *P* < 0.001, *r* = 0.707), higher lactate (2.0 vs. 1.4 mmol/L, *P* < 0.001, *r* = 0.383), and higher inotropic score (8 vs. 5, *P* < 0.001, *r* = 0.190). Among these, RVOT diameter demonstrated the largest effect size (*r* = 0.714), indicating a strong association with delayed extubation. No significant differences were observed in gender, age, weight, or transannular patch use between the two groups (all *P* > 0.05).

**Table 1:** Baseline characteristics of the study population.

Variable	Delayed Group (*n* = 101)	Non-delayed Group (*n* = 88)	*U*/*χ*^2^	*P* value
Age (months)	12.0 (8.8, 24.0)	12.0 (7.7, 30.8)	4,025.0	0.260
Weight (kg)	9.8 (8.0, 11.0)	9.8 (7.7, 15.5)	4,051.0	0.295
SpO_2_ (%)	89.0 (82.0, 95.0)	94.0 (85.0, 97.0)	3,436.5	0.007*
RVOT (mm)	4.1 (3.5, 5.0)	5.0 (4.0, 6.1)	2,725.5	<0.001***
McGoon ratio	1.8 (1.6, 2.0)	1.9 (1.7, 2.1)	3,292.0	0.002**
Nakata index	194.0 (175.0, 243.0)	252.0 (194.0, 314.0)	3,169.5	<0.001***
CPB time (min)	57.0 (48.0, 70.0)	48.0 (40.0, 59.5)	5,962.5	<0.001***
Oxygenation index	246.0 (198.0, 310.0)	310.0 (263.0, 345.0)	2,796.0	<0.001***
Lactate (mmol/L)	2.0 (1.2, 2.7)	1.4 (1.0, 2.0)	5,889.0	<0.001***
Inotropic score	8.0 (6.0, 10.0)	5.0 (4.0, 6.0)	7,501.0	<0.001***
Postop PVOV (m/s)	2.4 (2.1, 3.2)	2.2 (1.9, 2.6)	5,411.5	0.006**
Male gender, *n* (%)	53 (52.5%)	47 (53.4%)	0.02	0.898
Transannular patch, *n* (%)	30 (30.0%)	28 (31.8%)	0.07	0.788

*P < 0.05.

**P < 0.01.

***P < 0.001

### 3.2 Univariate logistic regression analysis

Univariate logistic regression identified 16 variables significantly associated with delayed extubation (*P* < 0.05): SpO_2_ (OR = 0.955, *P* = 0.017), VSD diameter (OR = 0.903, *P* = 0.035), RVOT diameter (OR = 0.593, *P* < 0.001), McGoon ratio (OR = 0.216, *P* = 0.006), Nakata index (OR = 0.994, *P* < 0.001), LVEDVI (OR = 0.958, *P* = 0.045), CPB time (OR = 1.042, *P* < 0.001), postoperative left atrial pressure (OR = 1.223, *P* = 0.003), oxygenation index (OR = 0.992, *P* < 0.001), lactate (OR = 1.922, *P* < 0.001), postoperative hemoglobin (OR = 0.972, *P* = 0.009), postoperative PVOV (OR = 1.833, *P* = 0.006), inotropic score (OR = 2.013, *P* < 0.001), AST (OR = 1.005, *P* = 0.022), total bilirubin (OR = 1.017, *P* = 0.030), and direct bilirubin (OR = 1.044, *P* = 0.035). Using a *P* < 0.10 threshold, 20 variables were advanced to LASSO regression. The inotropic score demonstrated the strongest univariate association (OR = 2.013 per unit increase), indicating that each point increase in inotropic score approximately doubles the odds of delayed extubation.

### 3.3 LASSO regression variable selection

**Figure 2:**
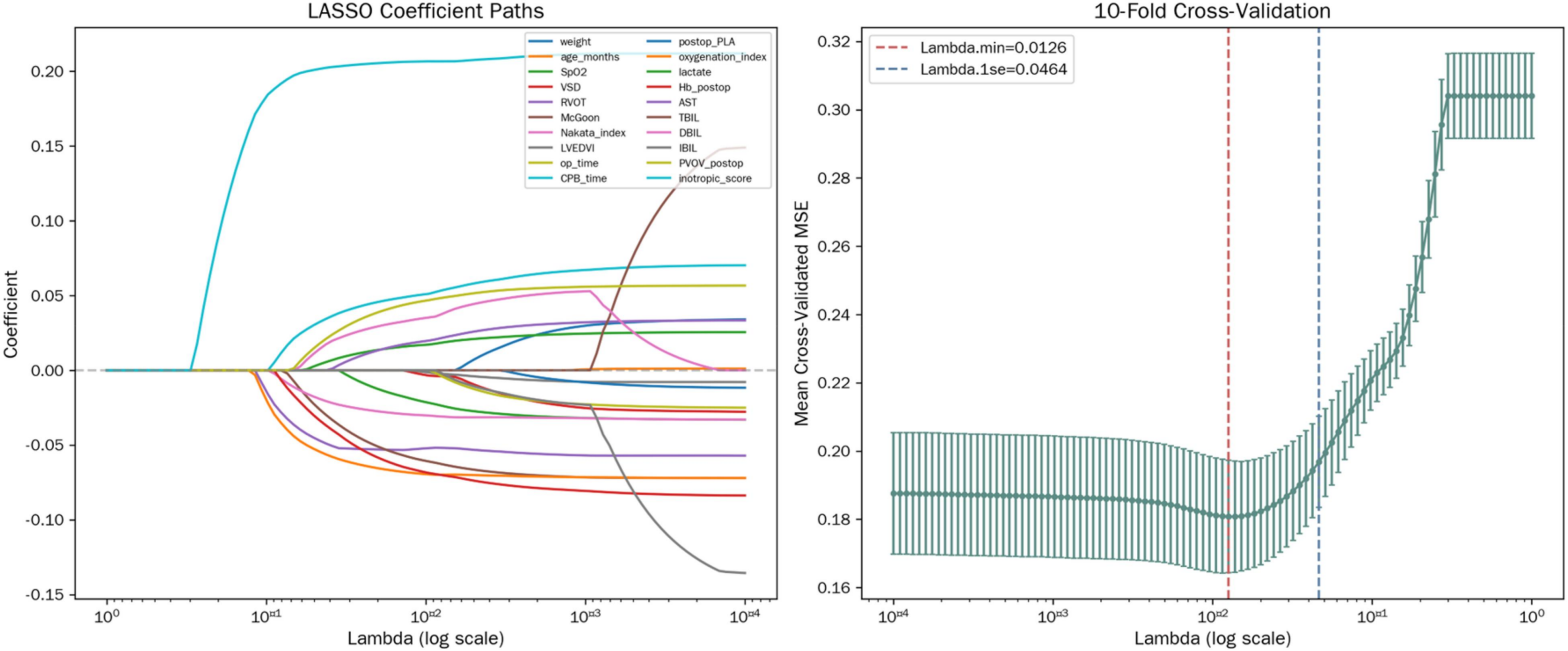
LASSO coefficient path diagram and 10-fold cross-validation.

LASSO regression with 10-fold cross-validation was applied to the 20 candidate variables. The optimal regularization parameter (lambda.1se) was selected using the 1-standard error rule, which favors model parsimony. Nine variables retained non-zero coefficients after LASSO shrinkage, ranked by absolute coefficient magnitude: inotropic score, oxygenation index, RVOT diameter, CPB time, McGoon ratio, postoperative hemoglobin, postoperative PVOV, Nakata index, and lactate. The LASSO coefficient path diagram (Figure 2) illustrates the progressive shrinkage of variable coefficients with increasing regularization, demonstrating that inotropic score, oxygenation index, and RVOT diameter are the most robust predictors across different levels of regularization. Eleven variables were shrunk to zero, including SpO_2_, age, weight, VSD diameter, postoperative left atrial pressure, LVEDVI, operative time, AST, and bilirubin markers, indicating their limited independent contribution after accounting for correlations with other predictors.

### 3.4 Multicollinearity assessment

Variance inflation factor analysis of the 9 LASSO-selected variables confirmed the absence of significant multicollinearity (all VIF < 1.3, well below the threshold of 5). The highest VIF was observed for inotropic score (VIF = 1.214), followed by CPB time (VIF = 1.138) and RVOT diameter (VIF = 1.106). These results indicate that the selected predictors provide largely independent information, and the multivariate regression coefficients will be stable and interpretable. The low VIF values also validate the LASSO regression approach, which effectively handles correlated predictors through regularization.

### 3.5 Multivariate logistic regression

The 9 LASSO-selected variables were entered into a multivariate logistic regression model. Backward elimination sequentially removed lactate, Nakata index, and postoperative hemoglobin, yielding a final model with 6 independent risk factors (Table 2). The model demonstrated strong overall fit: likelihood ratio test *χ*^2^ = 119.491 (*P* < 0.001), McFadden pseudo-*R*^2^ = 0.468, AIC = 150.06, and BIC = 172.60.

**Table 2:** Multivariate logistic regression analysis of risk factors for delayed extubation.

Variable	Coefficient (SE)	Wald	OR	95% CI	*P* value
Inotropic score	0.693 (0.128)	29.102	1.999	1.554–2.571	<0.001
Oxygenation index	−0.010 (0.003)	10.138	0.990	0.984–0.996	0.002
RVOT diameter	−0.341 (0.153)	4.965	0.711	0.527–0.960	0.026
CPB time	0.027 (0.014)	3.870	1.027	1.000–1.055	0.049
McGoon ratio	−2.170 (0.834)	6.765	0.114	0.022–0.586	0.009
Postop PVOV	0.667 (0.313)	4.536	1.947	1.055–3.596	0.033

The six independent risk factors, organized by temporal phase, were: (1) Preoperative phase: McGoon ratio (OR = 0.114, 95%CI: 0.022–0.586, *P* = 0.009) and RVOT diameter (OR = 0.711, 95%CI: 0.527–0.960, *P* = 0.026); (2) Intraoperative phase: CPB time (OR = 1.027, 95%CI: 1.000–1.055, *P* = 0.049); (3) Postoperative phase: inotropic score (OR = 1.999, 95%CI: 1.554–2.571, *P* < 0.001), oxygenation index (OR = 0.990, 95%CI: 0.984–0.996, *P* = 0.002), and postoperative PVOV (OR = 1.947, 95%CI: 1.055–3.596, *P* = 0.033). The inotropic score was the strongest predictor, with each unit increase associated with a 99.9% increase in the odds of delayed extubation. The McGoon ratio demonstrated the second strongest effect, with each unit increase associated with an 88.6% reduction in the odds of delayed extubation.

### 3.6 Model performance evaluation

#### 3.6.1 Discrimination: ROC curve analysis

**Figure 3:**
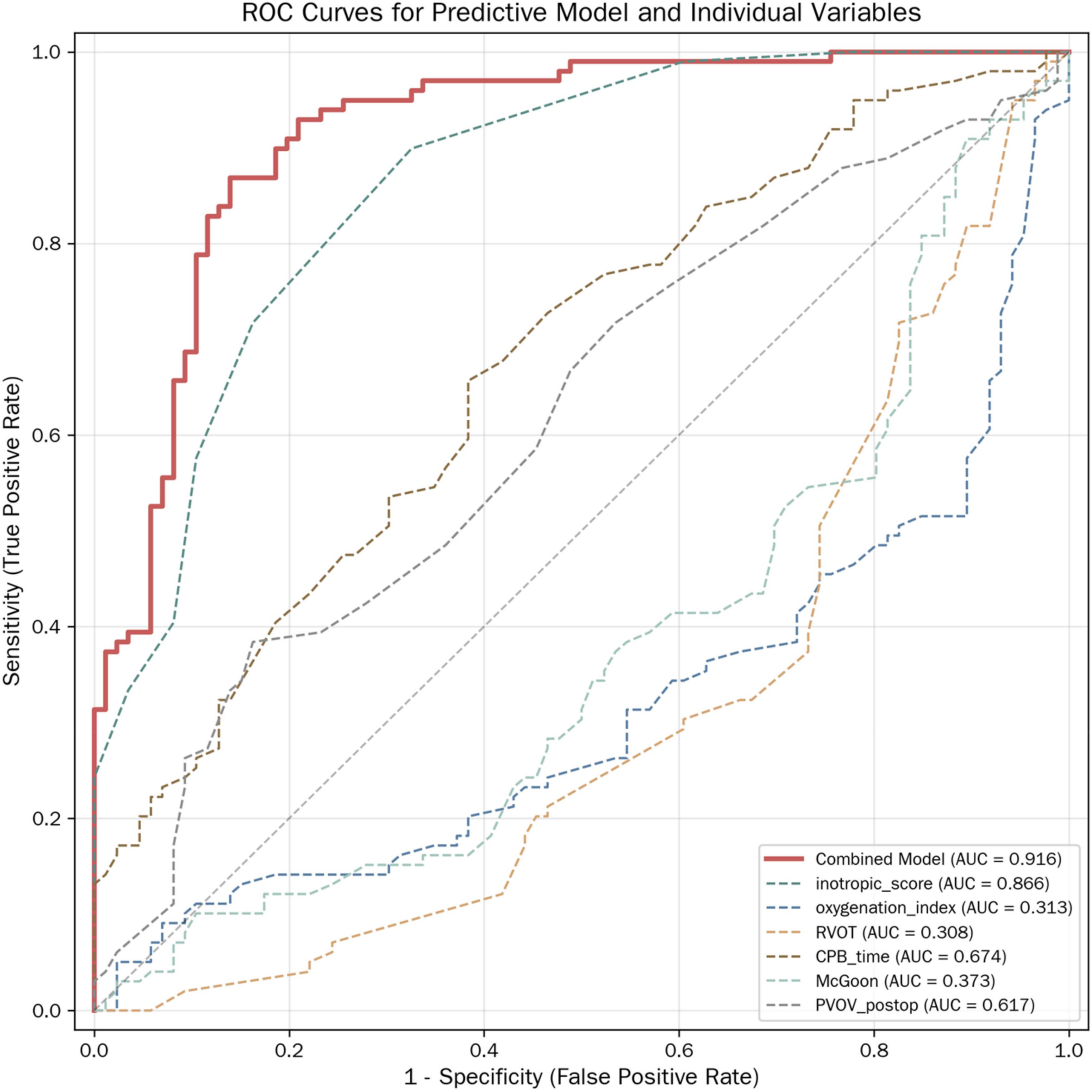
ROC curves for individual predictors and the combined model.

The ROC curve analysis (Figure 3, Table 3) demonstrated that the combined six-variable model achieved an AUC of 0.916, indicating excellent discrimination. At the optimal threshold determined by Youden index (*J* = 0.729), the model achieved a sensitivity of 86.9% and specificity of 86.0%. Among individual predictors, inotropic score had the highest standalone AUC (0.866, cut-off = 6.0, sensitivity = 89.9%, specificity = 67.4%), followed by CPB time (AUC = 0.674), postoperative PVOV (AUC = 0.617), oxygenation index (AUC = 0.687), RVOT diameter (AUC = 0.692), and McGoon ratio (AUC = 0.627). The combined model substantially outperformed any single predictor, confirming the synergistic value of integrating preoperative, intraoperative, and postoperative variables.

**Table 3:** ROC curve analysis for individual predictors and combined model.

Variable	AUC	Cut-off	Sensitivity	Specificity	Youden Index
Inotropic score	0.866	6.0	89.9%	67.4%	0.573
Oxygenation index	0.687	287.0	65.7%	62.8%	0.285
RVOT diameter	0.692	4.5	55.6%	76.7%	0.323
CPB time	0.674	52.0	65.7%	61.6%	0.273
McGoon ratio	0.627	1.8	55.6%	65.1%	0.207
Postop PVOV	0.617	2.8	38.4%	83.7%	0.221
Combined Model	0.916	0.507	86.9%	86.0%	0.729

#### 3.6.2 Calibration assessment

**Figure 4:**
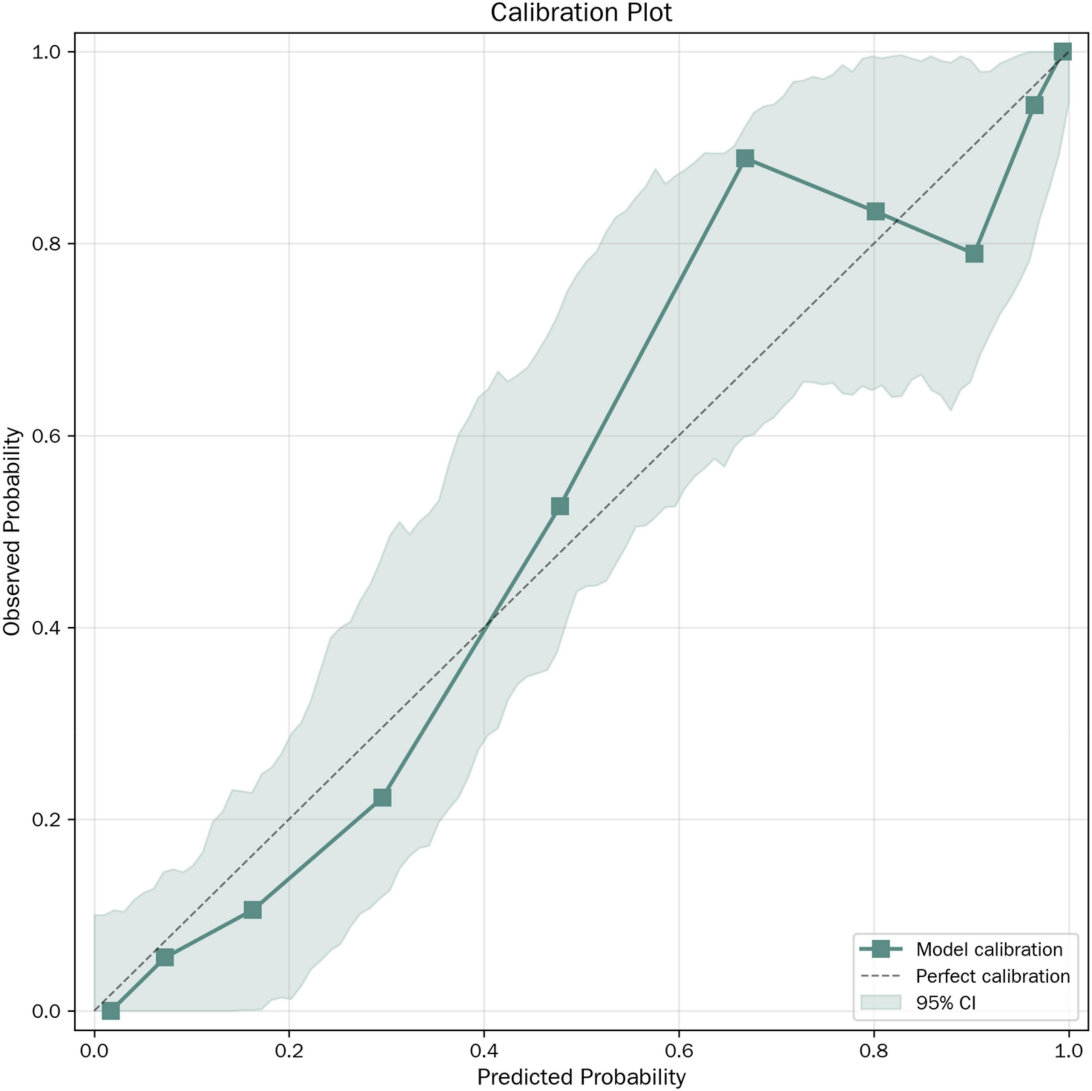
Calibration plot for the combined predictive model.

The calibration plot (Figure 4) demonstrated good agreement between predicted and observed probabilities across the range of predicted risks. The Hosmer–Lemeshow goodness-of-fit test yielded *χ*^2^ = 8.731 with *P* = 0.366, indicating no significant deviation from perfect calibration (*P* > 0.05 supports adequate fit). The Brier score was 0.113, substantially below the 0.25 threshold for a non-informative binary model, confirming that the model provides well-calibrated probability estimates. The combination of excellent discrimination (AUC = 0.916) and good calibration (H–L *P* = 0.366, Brier = 0.113) indicates that the model not only effectively distinguishes between delayed and non-delayed patients but also provides accurate probability estimates that can be trusted for clinical decision-making.

#### 3.6.3 Decision curve analysis

**Figure 5:**
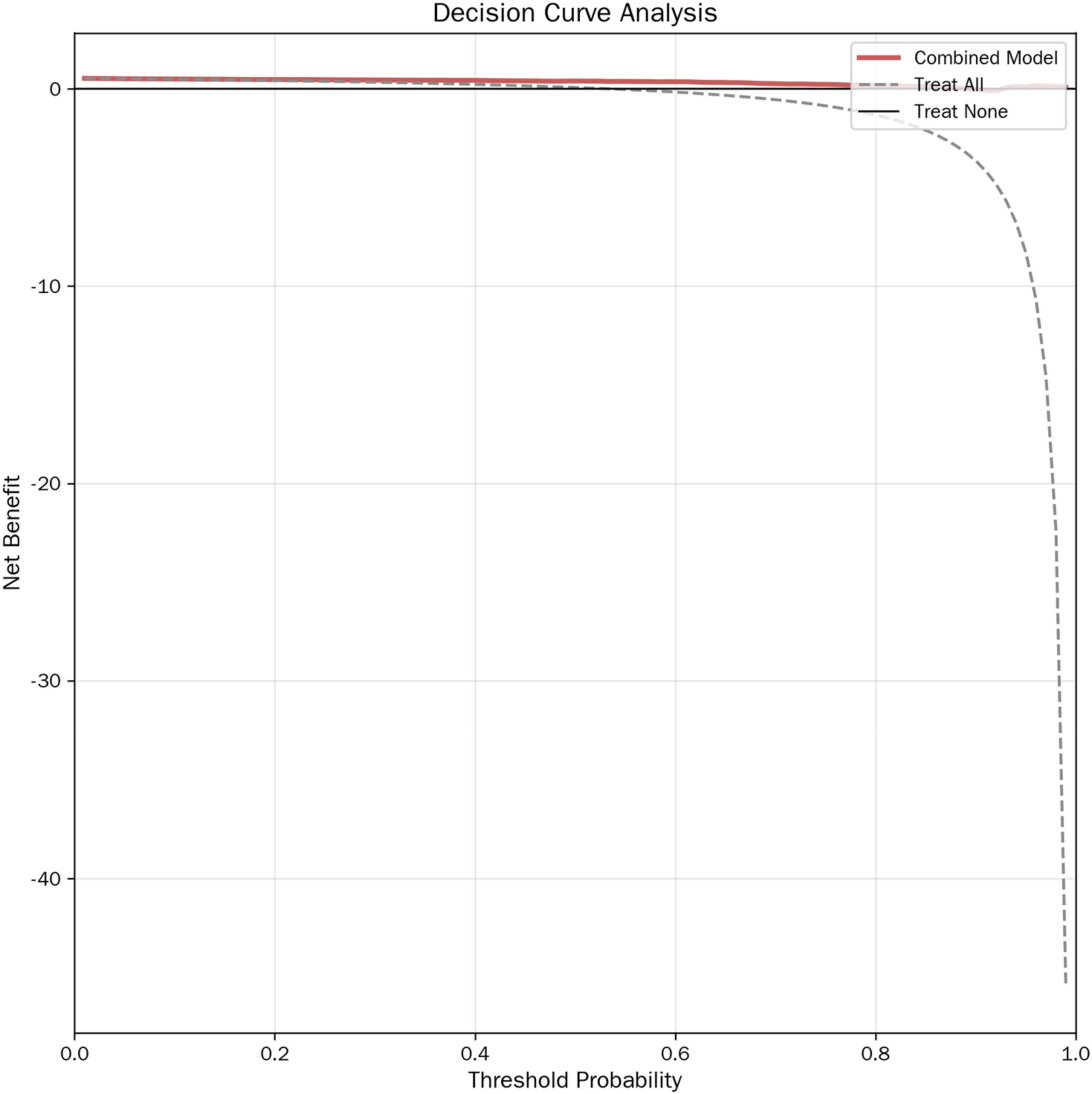
Decision curve analysis for the combined predictive model.

**Figure 6:**
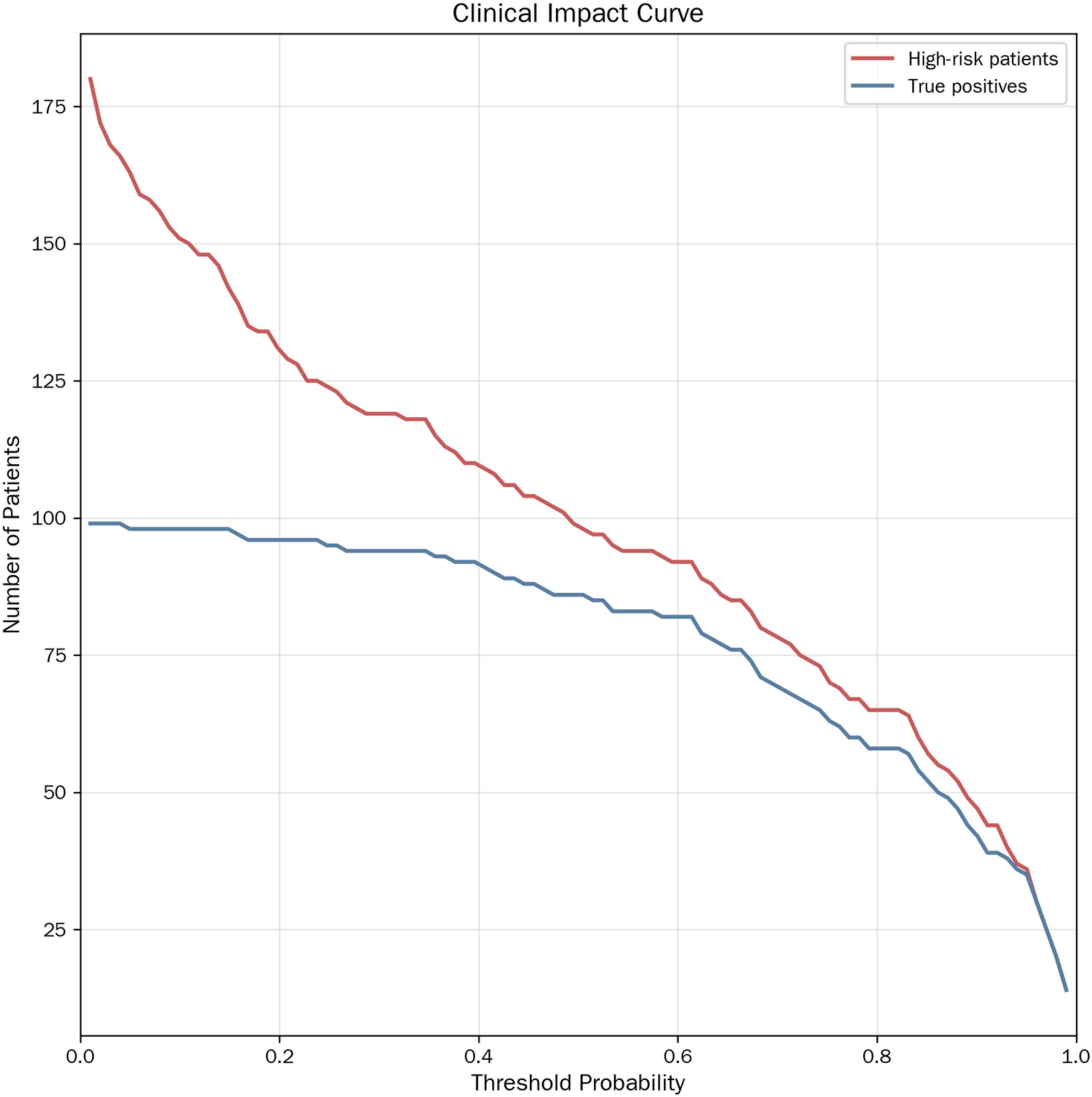
Clinical impact curve for the combined predictive model.

Decision curve analysis (Figure 5) demonstrated that the combined model provides net benefit superior to both the “treat-all” and “treat-none” default strategies across threshold probabilities ranging from approximately 10% to 85%. This range encompasses the clinically relevant decision thresholds for identifying high-risk patients who may benefit from targeted interventions such as aggressive pulmonary vasodilation, optimized fluid management, or early mobilization strategies. The clinical impact curve (Figure 6) further illustrated that at a threshold probability of 50%, approximately 95 patients would be classified as high-risk, of whom approximately 88% would be true positives, demonstrating favorable predictive efficiency.

### 3.7 Nomogram

**Figure 7:**
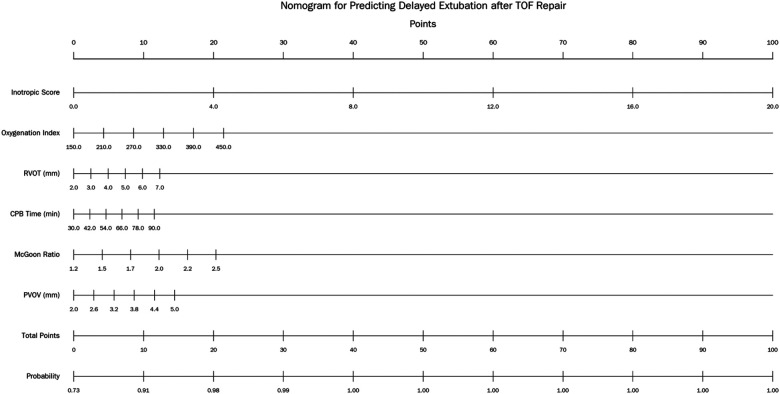
Nomogram for predicting delayed extubation after TOF repair.

A nomogram (Figure 7) was constructed based on the final six-variable model, enabling bedside risk prediction. Each predictor is assigned a point scale proportional to its regression coefficient: inotropic score (reflecting its largest coefficient), McGoon ratio, RVOT diameter, postoperative PVOV, CPB time, and oxygenation index. The total points are mapped to a predicted probability of delayed extubation ranging from less than 10% to greater than 90%. Clinicians can rapidly estimate individual patient risk by summing the points corresponding to each variable value, providing a practical tool for real-time clinical decision support.

### 3.8 Internal validation

**Figure 8:**
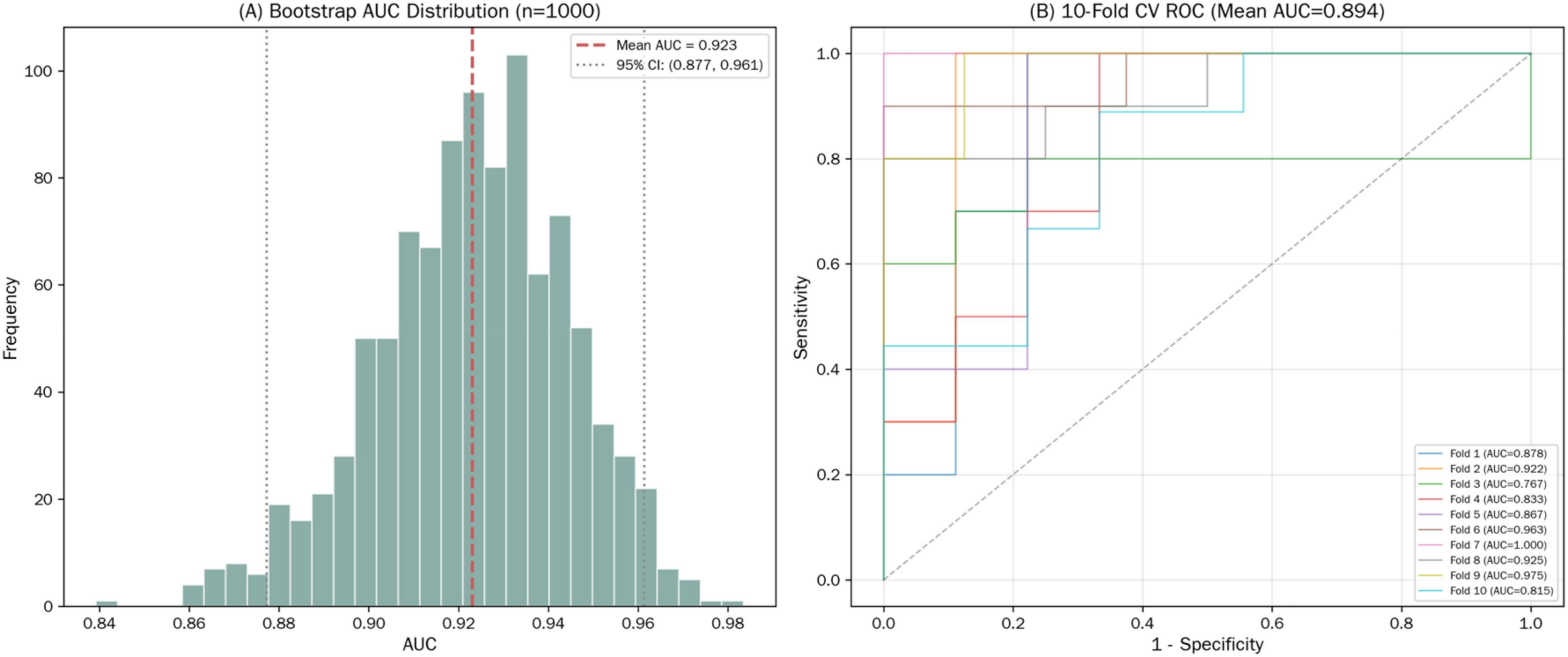
Bootstrap internal validation results.

Bootstrap internal validation with 1,000 iterations yielded a bootstrap mean AUC of 0.923 and an optimism of 0.007, resulting in a corrected AUC of 0.909 (95%CI: 0.877–0.961) (Figure 8). The minimal optimism indicates negligible overfitting. The bootstrap Brier score was consistent with the original estimate. Stratified 10-fold cross-validation yielded a mean AUC of 0.894 ± 0.072, with all folds achieving AUC > 0.80 (Figure 8). The concordance between bootstrap and cross-validation results, along with the minimal optimism, provides strong evidence for the model's internal validity within the study population. It is important to note that these results represent internal validation only; external validation in independent cohorts is essential before clinical implementation.

### 3.9 Subgroup analysis

**Figure 9:**
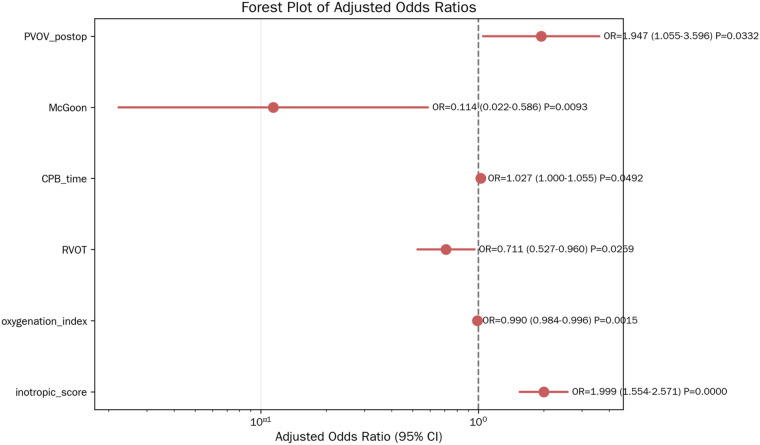
Forest plot of adjusted odds ratios from the multivariate logistic regression model.

Subgroup analysis (Table 4) demonstrated consistent model performance across key patient subgroups (Figure 9). The model AUC was similar between age subgroups (>12 months: AUC = 0.934; ≤12 months: AUC = 0.923), weight subgroups (≤10 kg: AUC = 0.926; >10 kg: AUC = 0.929), and transannular patch subgroups (with patch: AUC = 0.953; without patch: AUC = 0.914). The delayed extubation rate was higher in younger patients (≤12 months: 58.5% vs. > 12 months: 44.8%) and lower-weight patients (≤10 kg: 57.1% vs. > 10 kg: 47.9%), consistent with the known vulnerability of smaller infants to postoperative respiratory complications. The consistent model discrimination across subgroups suggests that the predictive model is robust within the study population.

**Table 4:** Subgroup analysis of model performance.

Subgroup	N	Delayed, *n* (%)	AUC	95% CI
Age >12 months	67	30 (44.8%)	0.934	—
Age ≤12 months	118	69 (58.5%)	0.923	—
Weight ≤10 kg	112	64 (57.1%)	0.926	—
Weight >10 kg	73	35 (47.9%)	0.929	—
With Patch	57	29 (50.9%)	0.953	—
Without Patch	127	69 (54.3%)	0.914	—

### 3.10 Sensitivity analysis

**Figure 10:**
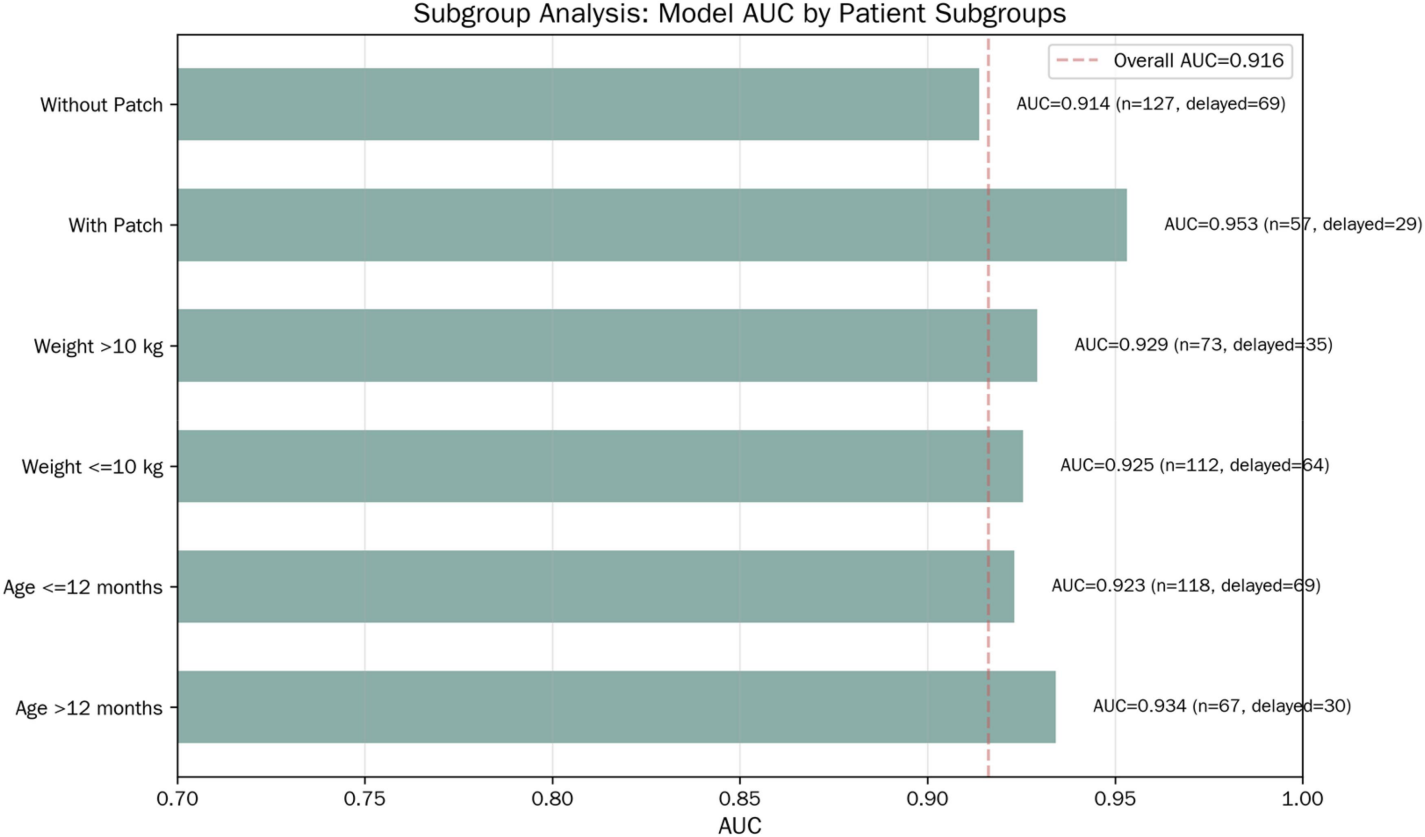
Subgroup analysis showing model AUC across different patient subgroups.

**Figure 11:**
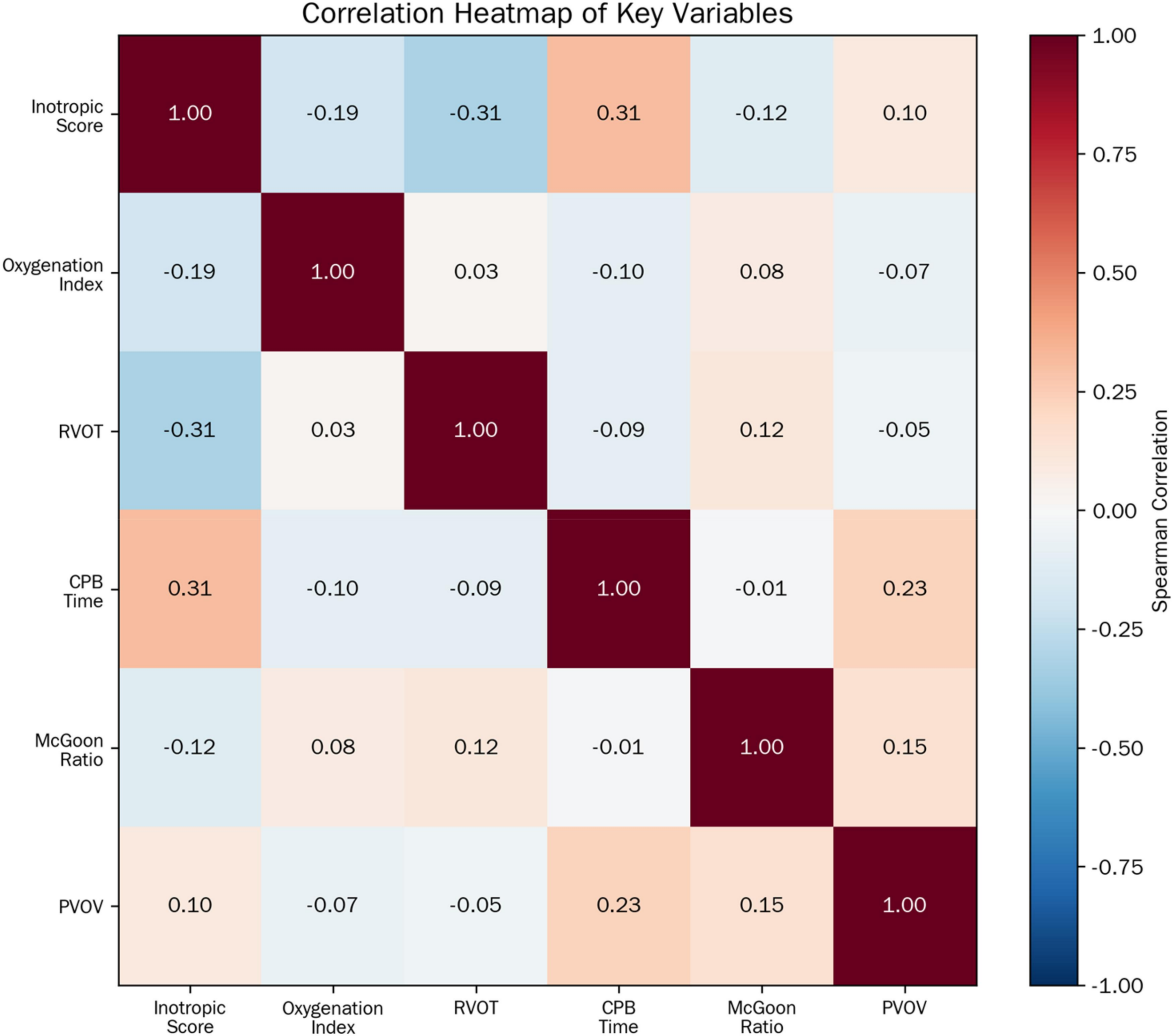
Correlation heatmap of key variables.

Leave-one-variable-out sensitivity analysis (Table 5) identified inotropic score as the most influential predictor, with its removal causing the largest AUC decrease (from 0.916 to 0.824, ΔAUC = 0.092) (Figure 10). McGoon ratio (ΔAUC = 0.010) and oxygenation index (ΔAUC = 0.017) were the next most influential predictors, while RVOT diameter (ΔAUC = 0.007), CPB time (ΔAUC = 0.006), and postoperative PVOV (ΔAUC = 0.004) had relatively smaller individual contributions. The relatively modest AUC decreases upon removal of individual variables (except inotropic score) suggest that the model is not overly dependent on any single predictor, supporting its robustness. Analysis using different *P*-value thresholds for univariate inclusion (*P* < 0.05, *P* < 0.10, *P* < 0.15, *P* < 0.20) yielded identical final models with 6 variables, demonstrating that the model is insensitive to the initial variable screening criterion and that the LASSO-backward elimination pipeline effectively identifies the core predictors regardless of the entry threshold (Figure 11).

**Table 5:** Leave-one-variable-out sensitivity analysis.

Variable Removed	AUC	ΔAUC
None (Full Model)	0.916	—
Inotropic score	0.824	0.092
Oxygenation index	0.900	0.017
McGoon ratio	0.906	0.010
RVOT diameter	0.910	0.007
CPB time	0.910	0.006
Postop PVOV	0.913	0.004

## 4 Discussion

Tetralogy of Fallot (TOF) is the most common cyanotic congenital heart disease in children, and surgical correction remains the only definitive treatment (18, 19). Although advances in surgical techniques have significantly improved long-term survival rates after TOF repair, surgery performed under cardiopulmonary bypass (CPB) involves substantial trauma and prolonged duration, leading to high perioperative risks (20, 21). Prolonged mechanical ventilation, a major complication, predisposes patients to adverse outcomes such as ventilator-associated pneumonia and respiratory muscle weakness, serving as an important cause of postoperative mortality. Given the narrow airways, poor respiratory reserve in children, and the pre-existing limitations of pulmonary blood flow and chronic hypoxia in TOF patients, these individuals are particularly susceptible to ventilator dependency (22–24).

Building upon this clinical context, the present study establishes a triphasic predictive framework for delayed extubation following primary TOF repair, integrating preoperative anatomical substrates, intraoperative physiological insults, and postoperative hemodynamic status. By employing rigorous statistical methodologies—specifically LASSO regression coupled with variance inflation factor analysis and dual internal validation—six independent predictors were identified: the McGoon ratio, RVOT diameter, CPB time, inotropic score, oxygenation index, and postoperative PVOV. The resulting model demonstrated exceptional discrimination (AUC = 0.916) and calibration, outperforming single-parameter assessments and providing a potentially clinically actionable nomogram for bedside risk stratification.

The identification of these six factors constructs a coherent pathophysiological cascade associated with ventilator dependency. Preoperatively, a lower McGoon ratio and smaller RVOT diameter are associated with underlying pulmonary vascular hypoplasia and right ventricular outflow tract obstruction. These anatomical substrates predispose patients to restricted pulmonary blood flow and elevated right ventricular afterload even after surgical correction. Intraoperatively, prolonged CPB time often reflects a higher degree of surgical complexity. Together, the more intricate surgical repair and the extended CPB duration act as surrogates for increased physiological insult, triggering systemic inflammatory responses and ischemia-reperfusion injury that contribute to postoperative capillary leak and pulmonary dysfunction (25, 26). Postoperatively, the persistence of high PVOV suggests residual outflow tract obstruction or significant regurgitation, sustaining right ventricular strain (27, 28). Crucially, the postoperative inotropic score emerged as the strongest predictor (OR = 1.999), quantitatively reflecting low cardiac output syndrome (LCOS) and the state of myocardial recovery. The interdependence of these phases suggests a complex interplay preoperative anatomical vulnerabilities compounded by CPB-induced inflammation can exacerbate postoperative cardiac dysfunction (high inotropic score) and impaired gas exchange (low oxygenation index), ultimately delaying cardiorespiratory recovery.

It is important to emphasize that the postoperative variables in our model—inotropic score, oxygenation index, and postoperative PVOV—should be interpreted as composite markers reflecting the interplay of preoperative anatomical substrates, intraoperative physiological insults (particularly CPB duration and surgical complexity), and early postoperative hemodynamic status, rather than as purely postoperative care parameters. The inotropic score primarily reflects myocardial dysfunction stemming from CPB-related ischemia-reperfusion injury and right ventricular surgical manipulation, compounded by preoperative anatomical severity. The oxygenation index reflects pulmonary dysfunction arising from CPB-induced systemic inflammatory response superimposed on pre-existing pulmonary vascular hypoplasia. This composite nature has important implications for clinical interpretation: the model is designed for sequential perioperative risk stratification rather than standalone preoperative prediction. We acknowledge that specific postoperative care variables—such as fluid balance, sedation scores, pain management protocols, ventilator settings, and intubation route—were not included in the model. This represents a deliberate modeling choice, as our goal was to identify factors that predict delayed extubation risk using variables that are routinely and objectively measured in all patients. Future model refinement could incorporate these additional care variables to improve predictive accuracy.

Methodologically, this study addresses several limitations often seen in predictive modeling for pediatric cardiac surgery. The utilization of LASSO regression effectively handled the high dimensionality of clinical variables while preventing overfitting. Furthermore, by strictly classifying variables temporally and excluding outcome-related complications (such as low cardiac output syndrome defined clinically rather than via score, pleural effusion, or reintubation) from the predictor pool, circular reasoning was mitigated, ensuring the model predicts rather than merely reflects the outcome. The internal validation yielded minimal optimism (0.007), confirming that the model's performance is robust and within the study population and not merely a product of overfitting to this specific cohort. However, we emphasize that these validation results represent internal validation only; external validation using independent multi-center cohorts is essential before clinical implementation. Clinically, the proposed nomogram may offer a dynamic tool for individualized risk stratification across the surgical timeline. Preoperative identification of high-risk anatomy (low McGoon ratio, small RVOT) may allow surgeons to anticipate complications and plan tailored surgical strategies, such as the judicious use of transannular patches vs. valve-sparing techniques (29, 30). Intraoperative vigilance in minimizing CPB duration, where feasible, may attenuates subsequent pulmonary and systemic inflammatory injury (31, 32). In the postoperative period, the integration of the inotropic score and oxygenation index provides objective, quantitative thresholds that could support targeting of early mobilization and ventilator weaning protocols, potentially guiding interventions such as optimized fluid management, pulmonary vasodilation, or escalation of inotropic support to facilitate earlier extubation (33, 34).

Several limitations warrant careful consideration. First, the retrospective, single-center design inherently carries selection bias and limits generalizability. Our center's status as a high-volume regional referral center may influence patient characteristics and outcomes, and our findings may not be directly generalizable to centers with different surgical techniques, perioperative management protocols, or case mix. External validation using multi-center prospective cohorts is essential before widespread clinical adoption. Second, although the sample size (*n* = 189) met the typical events-per-variable criteria, larger datasets might uncover additional modest predictors or stabilize estimates in specific subgroups (e.g., neonates vs. older infants). Third, the binary definition of delayed extubation at 48 h, while clinically relevant and commonly used, may not capture the nuances of ventilator weaning trajectories; future studies could consider time-to-event (survival) analysis. Fourth, certain potentially relevant variables—such as genetic syndromes (e.g., 22q11 deletion), detailed advanced echocardiographic strain parameters, surgeon experience, intraoperative transfusion practices, sedation protocols, nutritional status, or novel biomarkers of inflammation and myocardial injury—were not uniformly available and thus excluded. These unmeasured variables may introduce residual confounding that could affect the observed associations. Fifth, while temporal validity in variables was accounted for, evolving surgical techniques and perioperative management protocols over the study period (2019–2023) may introduce practice drift, though the consistency across subgroups suggests stability. Finally, as an observational study, we cannot establish causal relationships between the identified factors and delayed extubation; the reported associations may be influenced by unmeasured confounders and confounding by indication, particularly for postoperative variables such as the inotropic score.

This study contributes to the existing literature in several important ways. Previous studies by Faerber et al. employed machine learning approaches to predict complicated postoperative courses in TOF using the STS database (20), while Mahle et al. analyzed early extubation patterns in large multicenter cohorts (12). Our study differs from and extends this work by: (a) focusing specifically on delayed extubation as a primary outcome rather than as a component of composite endpoints; (b) integrating preoperative anatomical, intraoperative, and postoperative physiological variables in a single unified prediction model; (c) employing LASSO regression for variable selection in this specific clinical context; and (d) providing a clinically applicable nomogram with comprehensive decision curve analysis to facilitate bedside risk assessment. This integrated approach may provide clinicians with a more nuanced understanding of perioperative risk factors for ventilator dependency in TOF repair.

## 5 Conclusions

This analysis identified six independent risk factors associated with delayed extubation after primary TOF repair: McGoon ratio, RVOT diameter, CPB time, inotropic score, oxygenation index, and postoperative PVOV. The combined predictive model demonstrated excellent discrimination (AUC = 0.916) with good calibration (H–L *P* = 0.366), and internal validation confirmed its robustness within the study population (bootstrap-corrected AUC = 0.909). The triphasic risk model spanning preoperative, intraoperative, and postoperative phases provides a pathophysiologically coherent framework for understanding ventilator dependency after TOF repair. The nomogram and decision curve analysis suggest potential clinical utility for individualized risk prediction. However, several important caveats must be emphasized: (1) this model has undergone internal validation only and requires external validation in independent multi-center cohorts before clinical adoption; (2) as a retrospective single-center study, the findings may be subject to selection bias and limited generalizability; (3) causal relationships cannot be established from these observational data; and (4) prospective clinical implementation studies are needed to evaluate the model's impact on patient management and outcomes. Future research should focus on external validation, development of preoperative-only prediction models for earlier risk assessment, and exploration of additional postoperative care variables to further refine predictive accuracy.

## Data Availability

The raw data supporting the conclusions of this article will be made available by the authors, without undue reservation.
